# Producing the neoliberal city: spatial fragmentation and the politics of space in Batam, Indonesia

**DOI:** 10.3389/fsoc.2026.1788028

**Published:** 2026-06-17

**Authors:** Zeffry Alkatiri, Reynaldo De Archellie, Nazaruddin Nazaruddin

**Affiliations:** 1 Department of History, Faculty of Humanities, Universitas Indonesia, Depok, Indonesia; 2 Department of Area Studies, Faculty of Humanities, Universitas Indonesia, Depok, Indonesia; 3 Department of Linguistics, Faculty of Humanities, Universitas Indonesia, Depok, Indonesia

**Keywords:** differential space, migrant labour, spatial fragmentation, special economic zone (SEZ), state-corporate hegemony

## Abstract

Although Special Economic Zones (SEZs) have become a distinctive mode of neoliberal urbanisation across Southeast Asia, critical urban scholarship has rarelybrought Henri Lefebvre’s theory of the production of space into sustained dialogue with the concept of neoliberal space fragmentation in state-capitalist contexts in the Global South. This paper addresses that gap through an in-depth case study of Batam City, Indonesia. Established in 1973 as a state-planned industrial zone intended to rival Singapore, Batam has evolved into a paradigmatic site of neoliberal urbanisation in Southeast Asia. Drawing on 4 weeks of fieldwork (8–29 July 2024), eighteen semi-structured in-depth interviews analysed through NVivo-assisted thematic coding, and systematic triangulation with policy and planning documents, the study examines how spatial governance in Batam is shaped by the intersection of state authority, transnational capital, and everyday social practices. The findings show that Batam’s urban development reflects the dominance of abstract space—a technocratic and commodified spatial logic that privileges accumulation and efficiency over social needs. State–corporate alliances and overlapping bureaucracies have produced a fragmented spatial order in which industrial enclaves, infrastructure corridors, and exclusive residential zones coexist with informal worker settlements (*rumah liar*). These informal areas, though marginalised, constitute differential spaces that resist the homogenising tendencies of neoliberalisation by asserting alternative forms of inhabitation and community. By situating Batam within the broader dynamics of neoliberal urbanisation and state restructuring, the paper contributes to critical urban geography debates on how capitalist spatial production unfolds in semi-peripheral contexts. It argues that Batam exemplifies the material and ideological processes through which neoliberal hegemony is constructed, reproduced, and contested through space.

## Introduction

The debate on “Who has the right to the city?” is deeply intertwined with issues of urban transformation, gentrification, and spatial justice (Habibi et al., 2021). Scholars such as Harvey (2008) and Lefebvre (1991) have significantly contributed to this discourse, emphasising the need for inclusive urban spaces that serve all residents, particularly marginalised groups (Peck and Tickell, 2002). Originating from Lefebvre, the concept advocates for the collective right of urban inhabitants to shape and use urban spaces (Sechi and Golubchikov, 2025). It challenges the commodification of urban spaces driven by neoliberal policies, which often prioritise economic gains over social needs (Peck and Tickell, 2002; Schwake and Staničić, 2024). These dynamics are not merely economic but deeply political, as they reflect a struggle over whose interests define the urban future in a more democratic manner (Purcell, 2022). Power contestation occurs not only between residents and developers but also within state structures, where public policy often reinforces exclusionary spatial logics. Reclaiming the right to the city thus entails resisting neoliberal spatial governance and envisioning alternative urban futures grounded in equity and collective agency (Chan and Low, 2023). This paper positions itself within the strand of right-to-the-city scholarship that foregrounds spatial inequality and the lived experience of marginalised groups, rather than those strands that emphasise aesthetic or mobility-oriented readings of urbanism.

The significance of Lefebvre’s thinking on the production of space cannot be separated from the phenomenon of high economic growth in developing countries, particularly in Southeast Asia, following the global financial crisis of 2008. Studies by the Asian Development Bank (Lee and Hong, 2010), Silahtaroğlu et al. (2021), and di Clemente et al. (2021) show that the rapid growth of Southeast Asian countries is driven by factors such as human resources, investment, regulation, and, most importantly, the openness of economic policies (Trinh, 2024). Indonesia is one of the countries in Southeast Asia experiencing sustainable economic growth, with an average GDP growth rate of 4.8 percent since 2020 (World Bank, 2024), a factor attributed to the increasing influx of foreign investment. In the last 10 years (2014–2024), the Indonesian government has adopted an economic growth policy by building infrastructure using the dynamic cross-regional economic growth model (Badriah and Arintoko, 2024), one instrument of which is the Special Economic Zone (SEZ) mechanism.

The Indonesian government’s economic policies aim to boost foreign investment, alleviate poverty, and lower unemployment rates (Gupta, 2023; Dong and Manning, 2017). These goals are intended to enhance economic performance and improve the country’s competitiveness. To achieve this, the government has adopted a strategy focused on increasing exports, as noted by. Many countries typically encourage export growth by establishing Special Economic Zones (SEZs) or Free Trade Zones (FTZs), and Indonesia has followed suit. The government has pursued this approach through initiatives such as the ASEAN Free Trade Area, launched in 1992, and various bilateral agreements. More recently, Indonesia has developed multiple SEZs, a process that began between 2009 and 2012, with operations starting in 2015 (Mutia et al., 2019). One of the areas designated as a Special Economic Zone is the city of Batam, located in the western part of Indonesia, directly facing the Straits of Malacca and Singapore. Batam was established as a special authority area in 1973 as a Free Trade and Port Area. After the 1998 Reformation, Batam was granted autonomous status and strengthened with additional regulations in 2007 and 2009 as an industrial, trade, port, and tourism area. Since its opening as an industrial and trade area (1973–present), it has allowed various corporations and migrant workers from across Indonesia to come to work there, aiming to increase their social mobility.

Despite Batam’s evident importance as a Southeast Asian laboratory of neoliberal urbanism, a gap remains in the literature. While Lefebvrian analyses of the production of space have been extensively developed in post-socialist contexts (Batuman, 2019; Sechi and Golubchikov, 2025; Stanek, 2011) and Harvey’s (2012) critique has been mobilised across diverse cases, comparatively few studies have systematically applied these frameworks to SEZ-driven urbanisation in the Global South. Existing research on Batam (for example, Mutia et al., 2019) tends to adopt a developmentalist or policy-evaluation lens, documenting the zone’s economic performance without interrogating how its spatial order reproduces state–corporate hegemony and marginalises working-class inhabitants. This paper addresses that gap by examining the relationship between space as a political instrument and the mechanisms of power relations that shape spatial production to serve corporate interests, using Batam as a case study. Our focus is on how the production of space, driven by intersecting power dynamics, generates contestation among actors competing for control over strategic urban spaces. The complexities of spatial planning, production, and construction in Batam reflect its role as a site of negotiation, accommodation, and conflict, where diverse interests converge and compete in the ongoing struggle over space. It follows Lefebvre’s argument that spatial practice, representations of space, and representational spaces each play distinct roles in the production of space, shaped by their specific characteristics, the societal or production system in which they operate, and the historical context in which they emerge (Lefebvre, 1991, p. 46).

This study employs Lefebvre (1991) theory of the production of space to examine how power relations shape urban space in alignment with specific interests. Focusing on Batam City, it analyses spatial patterns that reveal ongoing contestation among actors involved in planning and development. In Lefebvre’s view, urban space is not a passive or neutral backdrop but a socially produced construct that actively reflects, reinforces, and legitimises dominant politico-economic orders. Through spatial practices, representations of space, and representational spaces, the urban environment becomes an arena where power is both materialised and contested. Accordingly, space functions simultaneously as a product of social relations and as a medium through which hegemonic interests are reproduced within a given mode of production. In the context of Batam, spatial conflict is thus not incidental but inevitable, as urban space tends to align with corporate and state-driven imperatives. Theoretically, this study contributes to Lefebvre’s concept of representational space by demonstrating how spatial meanings are actively produced, politicised, and contested through the interactions of state, corporate, and local actors in the lived experience of urban transformation.

## Conceptual framework

The theoretical foundation of this study is Henri Lefebvre’s well-known work, The Production of Space (1991), which conceptualises space as a socially produced and inherently contested phenomenon. Space is a product; any human knowledge of it must aim to uncover and articulate the process through which it is created. The investigation focuses on four dimensions: the actors, meaning who the producers are and who the objects are; the mode of production, referring to the historically specific ways in which societies organise, control, and shape space; ideology, understood as the underlying structure of the actors’ and producers’ designs of space; and actor interaction, concerning how the actors engage with one another when designing and constructing space (Lefebvre, 1991, pp. 35–36). The focus of inquiry must move beyond merely analysing objects within space to examining the very process by which space itself is produced, although this shift requires substantial clarification. Material objects and spatial discourses can only serve as indicators or evidence of this broader signifying process, which encompasses but cannot be reduced to these elements. In essence, our attention should be directed toward the productive processes through which space is actively shaped, involving an interplay between material forms, human knowledge, and discursive practices (Lefebvre, 1991, pp. 36–37).

According to Lefebvre, space becomes a tool used to create control and dominate others. In order to understand the perspective of space as a social product, Lefebvre developed a formulation called the conceptual triad of social space production, namely viewing social space dialectically by paying attention to (i) Spatial Practice, (ii) Representations of Space, and (iii) Representational Space. The Spatial Practice level encompasses the production and reproduction of community activities within a spatial context, which are reconstructed according to the interests of those in power, enabling them to transform them into social cohesion. This kind of spatial practice is understood as lived space, and from the analytic standpoint, the spatial practice of society is revealed through the deciphering of its space (Lefebvre, 1991, p. 38). Representations of Space, or conceived space, refers to the space of urban planners, scientists, architects, politicians, social engineers, and capitalists, all of whom identify what is lived and what is perceived with what is conceived. The conceptualisation of space tends to evolve into a system of verbal symbols that are intellectually constructed and articulated.

The third is Representational Space, which refers to space as it is directly lived and experienced through images, symbols, and everyday practices (Lefebvre, 1991, p. 39). It is the space of ordinary inhabitants and users, as well as some artists, writers, and philosophers who attempt to portray it without necessarily transforming it. This type of space is typically dominated and passively experienced, though the imagination often seeks to reinterpret or reclaim it. Generally, representational space tends to be expressed through relatively coherent systems of non-verbal signs and symbolic forms, though exceptions do exist, that abstract the existence of space as merely a commodity (Lefebvre, 1991, pp. 39–40). This space is also called perceived space. At this level, it becomes constructed because it has previously been conceptualised by several actors in the realm of conceived space. This formulation is a tool for examining how the hegemony of power relations regulates urban spatial planning as an object of capitalist commodities.

Lefebvre suggests analysing the dialectical relationship between these three concepts in order to understand how space is formed and constructed, particularly in capitalist societies (Lefebvre, 1991, pp. 39–41). It is plausible to suggest that spatial practice, representations of space, and representational space each play distinct roles in the production of space, shaped by their specific characteristics, qualities, and attributes, the prevailing societal or economic system, and the historical context in which they operate. Since Lefebvre’s original formulation, the framework has been significantly expanded. Brenner and Elden (2009) and Brenner (2019) extend Lefebvrian theory toward questions of scale and state-space, demonstrating how state restructuring actively produces the territorial configurations of capitalism. Goonewardena et al. (2008) resituate Lefebvre within a broader critique of everyday life, while Merrifield (2011, 2013) offers a Lefebvrian reconceptualisation of the right to the city that foregrounds ongoing practices of appropriation. Stanek (2011) and Schmid (2022) provide comprehensive syntheses that stress the dialectical and open-ended character of the triad. Because our case involves an SEZ whose design was explicitly modelled on large-scale industrial estates, it is also useful to recall Jacobs (1961) classic critique of modernist planning, which demonstrated how rigid zoning and monofunctional districts suppress the mixed-use vitality on which urban life depends. This pattern resonates strongly with the Batam case.

Recent critiques of neoliberalism in post-socialist contexts, especially those grounded in political science orthodoxy, have highlighted the discrepancies between neoliberal ideals and the actual economic structures of post-socialist states, pointing to the persistent influence of non-market and state forces. Yet, as Sechi and Golubchikov (2025) argue, such observations do not invalidate the continuing epistemological and ontological significance of neoliberalisation as the key process shaping contemporary urban transitions. Drawing on Lefebvre’s concept of the “levels of social practice,” they demonstrate how neoliberalism manifests through spatial fragmentation that reconfigures the socialist legacy of integrated welfare infrastructures into differentiated, commodified, and privatised urban landscapes. This fragmentation is not a by-product but an active mechanism that embeds neoliberal “common sense” within everyday life, linking macro-level ideological transformations to micro-level practices of housing, governance, and consumption. In this perspective, neoliberalisation should not be understood merely as market liberalisation or the withdrawal of the state, but as a broader hegemonic project that legitimises competition, entrepreneurialism, and commodification as normative social principles. While Batuman (2019) and Rutanen (2017) previously highlighted the interplay between spatial restructuring and ideological realignment in post-socialist cities, Sechi and Golubchikov’s approach advances the discussion by situating these processes within a Lefebvrian ontology of space—one that interprets urban fragmentation as both symptom and instrument of neoliberal hegemony. Hence, even amid hybrid or state-capitalist regimes, neoliberalisation persists as the dominant structuring logic of urban governance and everyday spatial experience.

Lefebvre’s triadic model, comprising spatial practice (perceived space), representations of space (conceived space), and representational spaces (lived space), provides a robust lens for reading Batam’s rapid urban transformation. Building on Lefebvre, Sechi and Golubchikov (2025) conceptualise neoliberalisation as an active project of space fragmentation: a spatial-ideological process that breaks down integrated welfare or collective urban forms into commodified, privatised fragments where neoliberal “common sense” is internalised in everyday practices. This fragmentation operates hand-in-glove with neoliberal urbanisation, transnational capital, and state-led spatial restructuring, intensifying spatial contestation and exclusion (Brenner and Elden, 2009; Stanek, 2011; Goonewardena et al., 2008; Schmid, 2022). In Batam’s free-trade zone, state policy produces privileged industrial, logistics, and commercial zones for capital accumulation while displacing and marginalising migrant settlements, creating a clear spatial hierarchy that reproduces precarity. Crucially, Sechi and Golubchikov’s framing shows that such reordering is not neutral technical planning but part of the capitalist production of space that relies on dispossession, enclosure, and spatial injustice, reinforcing Lefebvre’s critique of space as commodified (Harvey, 2012; Merrifield, 2011). Thus, Batam exemplifies how ideology, governance, and everyday life converge under neoliberal fragmentation to spatialise inequality.

Space, as Lefebvre argues, is socially produced through historical, social, and economic conditions rather than a neutral backdrop. Under capitalism, it functions as an instrument of accumulation, control, and commodification, embedding structural violence through processes of abstraction and enclosure. Yet differential space resists such homogenisation, defending use values and opening possibilities for alternative spatial practices. In this study, Lefebvre’s triadic model, comprising spatial practice, representations of space, and representational spaces, is applied to Batam’s urban transformation to reveal how state-corporate hegemony organises industrial zones and labour spatialities through neoliberal logics of fragmentation. By engaging recent debates on neoliberal urbanisation, transnational capital, and financialised space, the analysis extends Lefebvre’s insights and situates Batam within broader processes of spatial contestation and exclusion. Ultimately, Batam exemplifies the socio-political struggles inscribed in contemporary capitalist spatial production, where the pursuit of accumulation continually collides with claims for social justice and the right to space.

## Methods

This study adopts a qualitative research design to investigate the dynamics of spatial contestation in Batam City, focusing on how urban space is produced, organised, and contested among diverse actors. The theoretical approach is grounded in Lefebvre (1991) conception of space as socially produced, shaped by the interaction of spatial practices, representations of space, and representational spaces. This framework informs both the research questions and methodological strategy, enabling a critical analysis of how spatial forms reflect broader power relations, particularly under neoliberal urban conditions (Harvey, 2008; Merrifield, 2011). A single-case study strategy (Yin, 2018) was chosen because it allows in-depth, contextually grounded analysis of how urban space is produced and contested at a site that warrants sustained engagement rather than broad comparative treatment (Flyvbjerg, 2006). Batam was selected for four reasons: its historical depth as Indonesia’s longest-running SEZ, established in 1973; its distinctive dual governance structure, in which BP Batam operates alongside the elected Batam City Government; its transnational positioning within the Singapore–Johor–Riau growth triangle; and its empirical accessibility, which permitted entry into both state offices and informal settlements. The case covers Batam Island administered by BP Batam and the Batam City Government, with particular attention to the Batam Center administrative core, the Batamindo Industrial Park, the Tanjung Uncang shipyard cluster, middle- and upper-class residential enclaves, and the peripheral rumah liar settlements.

The object of analysis is the spatial transformation of Batam as a special economic zone, while the subjects include various stakeholders whose actions and interests shape or contest urban development. These range from state institutions and corporate actors to informal settlers, migrant labourers, and local experts. Data were collected through three complementary techniques: direct field observation, semi-structured in-depth interviews, and document analysis. Observation was conducted over 4 weeks (8–29 July 2024), through direct observation in the field to observe the characteristics of the research location, such as factory areas, warehouse areas, port areas, residential areas, business areas, and the suburbs of Batam City. Observations attended to four dimensions: the physical characteristics of the built environment; the social rhythms of daily life; visible markers of spatial inequality across zones; and interactions between formal and informal spatial practices. Each observation was recorded in structured field notes organised by date, site, duration, and observed dimension, and supplemented by geo-tagged photographs.

Interviews were conducted with eighteen purposively selected informants, chosen to represent the main stakeholder groups shaping Batam’s spatial production. Selection criteria included: decision-making authority or direct experience in at least one of the analytical sites; a minimum of 3 years’ active involvement in Batam’s economic, administrative, or community life; willingness to discuss politically sensitive questions on the basis of informed consent; and availability during the fieldwork window. Snowball sampling was used to reach informal settlers and migrant workers whose access required introduction by trusted community leaders. Informants comprised BP Batam officials (including the Head of Public Relations and technical staff), the Head of the Batam Land Office (BPN), businesspeople, senior journalists, community leaders, an urban observer, an ethnic-association leader, informal settlers, migrant workers, and local academics, including the director and deputy director of Politeknik Negeri Batam and a former chair of the Batam Regional People’s Representative Council. Semi-structured interviews are especially useful for exploring complex social phenomena while allowing flexibility in responses (Kvale and Brinkmann, 2015). Interview questions were designed to elicit both historical and spatial insights. Examples include: What is the history of Batam’s development as an economic zone since the 1970s? How did the government or managing authorities envision Batam as a Special Economic Zone? Which actors were involved in the design and planning of Batam’s spatial configuration? What international models or planning references were used in shaping Batam’s SEZ structure? How do ordinary inhabitants experience and negotiate the resulting spatial order?

The data collected in this qualitative study comprised (1) data on population, area, industrial area, and other city facilities; (2) data on issues, cases, and problems that have occurred over the last 10 years (2014–2024); (3) population data up to the first quarter of 2024, including the number of ethnic groups registered on the city government website; (4) data on the number of corporations in Batam up to 2023; and (5) data from direct field observations as eyewitnesses to issues and problems related to the contestation phenomenon in Batam. All interviews were audio-recorded with participants’ consent, transcribed verbatim, and analysed in NVivo 14 using the six-phase thematic coding procedure set out by Braun and Clarke (2006): familiarisation, generation of initial codes, searching for themes, reviewing themes, defining and naming themes, and producing the report. In the first cycle, codes were generated inductively from the interview transcripts, field notes, and documents. These initial codes were then clustered in NVivo 14 by grouping semantically related codes into thematic nodes. Clustering was guided by two criteria: empirical density (codes that recurred across multiple informants and sites were treated as candidates for a shared node) and conceptual affinity (codes describing similar social processes or spatial logics were merged into a single higher-order category). This produced an intermediate-level set of clustered nodes, for example, “land-lease tenure and UWTO payments,” “industrial zoning and infrastructure design,” “informal settlement practices and eviction cycles,” and “overlapping bureaucratic authority,” before any explicit theoretical labelling was applied.

In the second cycle, each clustered node was synchronised with Lefebvre (1991) conceptual triad: nodes pertaining to material urban infrastructure, zoning arrangements, and the spatial routines of workers and settlers were mapped onto spatial practice (perceived space); nodes capturing the discourse of planners, BP Batam officials, policy documents, and development blueprints were assigned to representations of space (conceived space); and nodes encoding the everyday experiences, informal survival strategies, and symbolic meanings expressed by residents, migrants, and community leaders were positioned within representational spaces (lived space). This synchronisation was not applied mechanically. Where a cluster of codes straddled more than one dimension of the triad, the node was linked to both dimensions and the tension was preserved as analytically significant. For example, an informant’s account of UWTO payments simultaneously described a material burden (spatial practice) and an experience of exclusion (representational space), and was therefore coded under both categories rather than forced into one. The resulting framework-mapped NVivo structure directly informed the organisation of the Findings section, with each subsection corresponding to one or more dimensions of the triad as they manifest in Batam’s spatial order: “Zoning, Infrastructure, and the Spatialisation of Inequality” draws primarily on nodes mapped to representations of space and spatial practice, while “Socio-Political Struggles of Batam’s Production of Space” draws on nodes mapped to representational spaces and their intersections with conceived space.

Triangulation followed Yin’s (2018) convergence logic, treating each of the three data strands as a separate source of evidence for every analytical claim. Where discrepancies arose between data strands, these tensions were retained and treated as analytically productive rather than resolved in favour of one source. One notable instance involved the contrast between official land-tenure narratives and informants’ lived experiences of UWTO payments. To strengthen validity, findings were also triangulated with secondary sources, including news articles, policy documents, planning regulations, and academic publications on Batam’s development (Yin, 2018). Preliminary findings were returned to three informants for member-checking before final write-up, and a 20 percent subsample of coded data was independently reviewed by a second coder (intercoder agreement *κ* = 0.81). This approach enables a nuanced understanding of Batam’s spatial politics and contributes to broader debates on urban governance and spatial justice under contemporary capitalist regimes.

## Findings: Batam and the production of space under state-corporate hegemony

Batam serves as a compelling case study for applying Lefebvre (1991) theory of the production of space, particularly in the context of state-led industrialisation and global capitalist integration. The island’s spatial formation reflects how state institutions, corporate actors, and transnational capital collaborate to shape urban development through regulatory and infrastructural interventions. BP Batam—the authority managing the island—controls spatial planning, land administration, and industrial zoning, while the Batam City Government regulates population and social affairs. This division of responsibility reflects a conceived space (representations of space) governed by technocratic rationalities and bureaucratic authority, where the state’s spatial vision is oriented toward sustaining capital accumulation. Fieldwork revealed that this division is not only administrative but also foundational to how Batam itself is imagined by those who produce it. The Head of the Batam Land Office, for example, actively resisted the researchers’ initial framing of the city as something built by a new government, offering instead a more revealing formulation:

*“Batam was not formed by a new government, sir, it was formed by new business. What came at that time was business, the government came afterwards. Back then, BP’s mission was indeed business—investment and so on. The government here was formed only several years after BP was already operating.”* (Head of BPN Batam, 9 July 2024)

This observation captures in plain language what Lefebvre (1991) describes as the violence of abstraction: a city conceived from the outset as an economic instrument rather than as a collective inhabited space. As an industrial and free-trade zone strategically located near Singapore, Batam has undergone rapid transformation driven by the intersecting powers of bureaucratic governance and private enterprise. The construction of industrial zones, labour settlements, and supporting infrastructure exemplifies Lefebvre’s notion that space is not a neutral container but a social product actively produced and organised through political and economic forces. Consistent with Sechi and Golubchikov’s (2025) concept of neoliberal space fragmentation, Batam’s spatial order reveals how neoliberalisation operates through the breaking apart of integrated urban forms into differentiated, commodified, and privatised spaces. This fragmentation not only reorganises the built environment but also embeds neoliberal “common sense” in everyday life—normalising competition, entrepreneurialism, and the responsibilisation of individuals and communities.

### Zoning, infrastructure, and the spatialisation of inequality

The creation of industrial areas, factories, and special economic zones in Batam reflects how space is produced to facilitate capital accumulation. Batam City is divided into zones. Zone I is located in the Batam Center area, which includes hotels, residential areas, ports, and government centres. Zone II, or the transition zone, includes squatter settlements, factories, and entertainment venues inhabited by various ethnic immigrants. Zone III is a residential area for workers in various formal sectors of the city. Zone IV is a middle-class residential area, with apartments and five-star hotels. Zone V is a suburban area inhabited by migrant workers close to industrial areas. Spaces that are constructed into physical spaces, such as industrial parks in Batam, indirectly show the form of inequality in the distribution of political power, social resources, and unequal urban infrastructure. All of this aims for the sustainability of capital accumulation, as justified by the formal legality in local regulations (Batam City Regional Smart City Masterplan 2023–2032; Batam City Regional Regulation No. 7/2021 concerning the Regional Medium-Term Development Plan 2021–2026). This spatial differentiation corresponds to what Lefebvre calls spatial practice—the material organisation of production, circulation, and consumption that reflects and sustains social hierarchies. Industrial parks such as Batamindo Industrial Park, established in 1990 through a Singapore–Indonesia joint venture, embody the state–corporate alliance that drives the island’s economy. Supported by ports, power plants, and roads, these sites exemplify the conceived and perceived spaces of neoliberal urbanism—designed for efficiency, export orientation, and the seamless movement of goods and labour.

Interview data reveal that this logic was inscribed in the city’s original design. The Head of BPN recalled his reading of the founding blueprint under B. J. Habibie, then Minister of Research and Technology:

*“According to Pak Habibie, Batam was the most potential place for us to go head to head economically with Singapore. So he came with economic plans. His aspiration was an economic aspiration. The blueprint I have seen and the city design are purely economic. Even at that time, housing was only designed to support the industries.”* (Head of BPN Batam, 9 July 2024)

This articulation, “housing was only designed to support the industries,” is a textbook instance of Lefebvre’s representations of space: planners and capitalists identifying what is lived and what is perceived with what is conceived. The veteran journalist-sociologist Socrates, who has covered the city since 1997, reached the same conclusion from the other side of the state–society relation:

*“They only thought about how to make Batam an economically profitable area. They forgot to address the social impacts on society. They did have a masterplan, but they never thought about what the consequences would be.”* (Socrates, former senior journalist of Batam Pos, 8 July 2024)

According to BP Batam (2023), there are 83 industrial estates hosting more than a thousand companies, most of them foreign-owned. Batam’s shipyard and port activities, concentrated in Sekupang, Batu Ampar, Kabil, and Tanjung Uncang, reinforce its role in regional and global production networks. This spatial configuration reflects the logic of neoliberal fragmentation described by Sechi and Golubchikov (2025): the city becomes a mosaic of functionally specialised enclaves serving transnational capital, while the social spaces of workers remain peripheral and precarious. The government’s establishment of new Special Economic Zones, including Batam Aero Technic, Nongsa Digital Park, and Sekupang Health Services SEZ, demonstrates the continued prioritisation of capital-oriented spatial restructuring. Extensive road and airport developments facilitate the circulation of goods, information, and labour, positioning Batam as a logistical and investment hub in competition with Singapore. The integration of SEZs with tourism and health services reflects the expansion of the commodified city, where even leisure and wellness are reorganised as components of the accumulation process. Business-facilitation policies, such as the Online Single Submission (OSS) system and tax-holiday schemes, further consolidate the state–corporate hegemony over urban space. By enabling transnational mobility through streamlined visa processes, these measures sustain Batam’s dual identity as an export-oriented manufacturing centre and a regional tourist destination. The resulting spatial order exemplifies how the neoliberal state functions not by withdrawing from intervention but by actively legitimising and structuring the commodification of space.

Tertiary education has been reorganised to fit this logic. The director of Politeknik Negeri Batam explained that the polytechnic was itself established in 2000 at the urging of BP Batam and the industries it hosted, with curricula designed to be, in the director’s phrase, “plug and play” with industrial demand, and with a one-year internship model that functions as a pipeline for corporate recruitment (Director, Politeknik Negeri Batam, 12 July 2024). Even human-capital formation is thus incorporated into the circuit of accumulation, further illustrating how comprehensively Batam’s urban fabric has been organised around the imperative of making the city legible and useful to global capital.

### Socio-political struggles of Batam’s production of space

While the conceived and material spaces of Batam serve global capital, the lived spaces of its residents reveal the contradictions of neoliberal urbanisation. Batam’s rapid industrialisation has attracted a vast influx of migrant labour from across Indonesia, producing a spatial division of labour manifested in distinct residential and social zones. Worker housing, typically low-cost dormitories or compact settlements, is situated near industrial estates to ensure labour circulation. In contrast, middle- and upper-class neighbourhoods, in which often inhabited by expatriates, enjoy superior infrastructure and amenities. This segregation exemplifies Lefebvre’s critique that capitalist production of space reinforces class hierarchies through spatial differentiation. The social costs of this arrangement are not merely distributive but existential. Socrates recalled a diagnosis offered by the late psychologist Sartono Mukadis that still circulates in Batam:

*“Batam is a city without a soul. It was simply not designed for people to live in comfortably. Life here is very hard.”* (Sartono Mukadis, cited by Socrates, 8 July 2024)

This evocative phrase captures, in vernacular form, precisely what Lefebvre meant by abstract space: a landscape fashioned as an economic instrument, legible to investors but alien to its inhabitants.

This study identifies two key issues related to spatial segregation in Batam City as an industrial urban area: (1) the ambiguous division of authority between the Batam City Government and BP Batam, the agency responsible for managing industrial zones, and (2) the proliferation of informal settlements or squatter housing, stemming from the municipal government’s limited capacity to manage land assets. The dual authority structure, that rooted in the historical tension between national control and local autonomy, creates what Sechi and Golubchikov (2025) call a fragmented institutional landscape. The coexistence of parallel bureaucratic hierarchies allows for negotiation and power brokerage in land allocation, often bypassing formal mechanisms. The Head of Public Relations of BP Batam acknowledged the institutional pathology in candid terms when recalling the pre-2019 period:

*“These two institutions, with two different leaderships, were clashing. Their policies overlapped, not synergistic. That is why in 2019 the central government decided: it is better if they are unified, so there is one command.”* (Head of Public Relations, BP Batam, 11 July 2024)

The secretary of the Batam branch of the PKB political party put the same point in the form of a local joke:

*“The mayor, as people used to say, 'had only the people’, while the Authority ‘had the land’, and that is still how it works today.”* (Secretary, PKB Batam, 12 July 2024)

The joke compresses the entire problem into two clauses. Formal local government has jurisdiction over inhabitants but not over the land on which those inhabitants live; BP Batam has jurisdiction over the land but no democratic accountability to the people who occupy it. As local informants explained, this ambiguity fosters uneven enforcement of land regulations, producing conditions ripe for conflict, opportunism, and inequality.

Historically, BP Batam has issued land-lease permits rather than full ownership titles, reinforcing residents’ dependence on state authority and limiting tenure security. The imposition of the Annual Obligation of Authority (Uang Wajib Tahunan Otorita or UWTO) further burdens residents with recurring payments, making legal land access unattainable for many low-income workers. Consequently, large numbers of migrants have turned to informal settlements or rumah liar (literally “wild houses”)—semi-permanent dwellings constructed on unregistered land. These settlements lack adequate infrastructure and public services but persist as necessary spaces of survival within Batam’s neoliberal spatial order. The existence of ruli communities epitomises Lefebvre’s notion of representational space: the lived, experienced, and contested realm where everyday practices resist or adapt to imposed spatial logics. Socially, these communities are segregated from formal residential areas; politically, their participation is instrumentalised during elections without meaningful improvement in living conditions. Their precarious legality and exposure to eviction threats reveal the deep contradictions of Batam’s developmental trajectory: an urban space produced to serve accumulation yet haunted by exclusion.

Strikingly, the Head of BPN described these settlements not as marginal to the city but as embedded in its very reproduction as an economic zone. Recalling the experience of relocated ruli residents, he observed:

*“We are all ruli, sir. We are evicted from the place we have made wild, and now we are made legal, ‘over there’. We carry our backpacks and live there. Then suddenly a friend comes by and, just casually, asks: ‘Do you want to buy my house?’ So the land I got for free, I sell it again. I take the money and go back to being a ruli. Some people here even consider ruli a profession.”* (Head of BPN Batam, 9 July 2024)

This remarkable passage, offered by a senior state official, discloses how abstract space and differential space are not two separate worlds but dialectically linked. Evictions and relocations do not eliminate informality; they metabolise it. The state produces commodified land, residents extract a one-time exchange value from their compensated plots, and the cycle resumes elsewhere in the urban periphery. Through this cycle, the formally illegal becomes functionally systemic. Viewed through Sechi and Golubchikov’s (2025) lens, Batam’s spatial order reflects neoliberal space fragmentation: the disassembly of collective social life into differentiated enclaves that naturalise inequality. Industrial parks, SEZs, and luxury zones coexist with informal settlements, forming a landscape of juxtaposed prosperity and marginality. This duality reveals how the state and capital jointly produce space as both a means of control and a site of contestation.

In sum, Batam’s development embodies the dialectical interplay of conceived, perceived, and lived spaces within a neoliberal framework. The state–corporate alliance, transnational investment networks, and fragmented urban governance together produce a spatial regime that privileges accumulation over equity. Yet within this fragmented order, everyday practices of survival, negotiation, and informal adaptation persist—signalling the enduring potential of differential space to resist homogenisation and reassert the social dimensions of urban life.

## The production of space and urban contestation in Batam

Henri Lefebvre’s theory of the production of space provides a critical lens for understanding the political economy of Batam’s urban development, where state-led industrialisation, transnational capital, and bureaucratic authority intersect to reorganise space under neoliberal rationalities. At the heart of Lefebvre (1991) framework lies the claim that space is not a neutral container for social action but a social product, one that is historically and materially constructed through modes of production and relations of power. Space, therefore, is both a product and an instrument of political economy, a terrain through which capitalism reproduces itself. As Batam’s trajectory illustrates, urban development is not a purely technical or administrative enterprise but a deeply political process in which different actors, ideologies, and institutional forces converge, conflict, and negotiate the right to produce and inhabit space.

Lefebvre’s guiding question—who produces space, and for whom?—is especially relevant in Batam’s context. The city’s spatial production is overwhelmingly shaped by institutional actors: the central government through BP Batam, domestic and foreign investors, and, to a lesser extent, the Batam City Government. These entities possess the legal, financial, and technical capacity to define land allocation, zoning, and development priorities. Their decisions determine whose spatial needs are prioritised and whose are marginalised. Ordinary residents, particularly low-wage migrant workers and informal settlers, occupy a peripheral position in this process: they are not co-producers of space but its subjects, inhabiting the margins of state–corporate designs. In Lefebvre’s terms, their lived spaces are subordinated to the representations of space—the bureaucratic, technocratic, and commercial logics that dominate spatial decision-making. This dynamic is reflected in the way Batam’s urban landscape has been conceived and managed. Spatial production is top-down, characterised by state–corporate coordination and minimal citizen participation. Planning documents, such as the Batam Smart City Masterplan (2023–2032) and the Regional Medium-Term Development Plan (2021–2026), prioritise industrial zoning, logistical infrastructure, and real-estate expansion aligned with investment attraction rather than social equity. The dominance of representations of space—plans, maps, and regulations—renders the everyday experiences of marginalised groups invisible, while their representational spaces, formed through lived practices, remain politically underacknowledged.

### Mode of production: neoliberal urbanisation and the violence of abstraction

For Lefebvre, the production of space under capitalism entails what he calls the violence of abstraction—the process through which lived, historically layered, and socially meaningful places are reduced to quantifiable commodities. This abstraction transforms land and built environments into assets circulating within capital’s logic of accumulation. Batam epitomises this process. The Head of BPN’s formulation that the city was founded “not by a new government but by new business” is, in theoretical terms, a description of abstract space at the moment of its institution: the priority of exchange value was not a later distortion of an initially social project but the project itself. Since its inception in the 1970s as an industrial and free-trade zone, the island has been spatially organised according to capitalist rationalities: efficiency, profitability, and global competitiveness. Infrastructure, zoning, and urban morphology are designed to facilitate the flow of goods, labour, and investment, a pattern that echoes what Sechi and Golubchikov (2025) describe as neoliberal space fragmentation.

This fragmentation refers not only to the physical disintegration of urban space into functionally specialised zones but also to the ideological normalisation of inequality. In Batam, industrial parks, logistics corridors, and Special Economic Zones embody the materialisation of abstract space. The Annual Obligation of Authority (UWTO) system institutionalises the commodification of land by converting access into a lease-based transaction, effectively excluding those who cannot afford to participate. The analytical significance of UWTO is that it operates simultaneously on two registers: materially, it transforms land from a lived resource into a recurring fiscal obligation; ideologically, it recodes land access as a matter of individual fiscal capacity rather than a social right, naturalising exclusion as a market outcome. Here, the state’s regulatory function aligns with market imperatives, enforcing an economic logic that privileges capital over community. The result is a spatial order that is both segmented and hierarchical, where accumulation and exclusion coexist as two sides of the same process.

Spatial segregation reinforces this abstraction. The city’s zoning structure, spanning from the administrative core in Batam Center to the industrial peripheries, embodies an uneven geography of privilege. Middle- and upper-class enclaves, often associated with expatriate and managerial populations, are endowed with infrastructure and amenities, while working-class and migrant communities are pushed to peripheral or informal zones. These disparities are not incidental but inherent to the capitalist production of space, where the pursuit of exchange value overrides the right to use and inhabit. As Lefebvre reminds us, capitalism produces not only commodities but also space as commodity, which serves simultaneously as a medium of accumulation and a means of control.

### Ideology and actor: developmentalism and the rationalisation of space

Lefebvre insists that space is also ideological: its production is guided by particular discourses that legitimise and naturalise domination. In Batam, this ideology manifests through a persistent developmentalist narrative that equates spatial modernisation with national progress. Developmentalism, with its focus on growth, infrastructure, and competitiveness, provides the ideological scaffolding that justifies large-scale spatial interventions. Under this discourse, space must be rationalised, disciplined, and rendered legible to investors. Industrial estates, bonded zones, and digital or health-focused SEZs are presented as symbols of modernity, while informal and communal spatial practices are framed as backward or inefficient. The striking element of our interviews is how openly state actors themselves articulate this developmentalist logic. The Head of BPN’s recollection that Habibie’s aspiration was explicitly “an economic aspiration” and that housing was designed “only to support the industries” is not a critical observation but a matter-of-fact description from inside the state apparatus. What is distinctive about Batam, in other words, is not that developmentalist ideology operates there, but that it is acknowledged without embarrassment because it has become common sense.

This ideology, as Sechi and Golubchikov (2025) argue, operates as a key component of neoliberalisation by transforming the state into an agent that produces and legitimises the commodification of space. Rather than withdrawing from intervention, the neoliberal state reorganises its functions to facilitate capital accumulation, rationalising inequality as the inevitable outcome of efficiency and competition. In Batam, this is visible in the state’s dual role: both as a regulator that controls land use and as an entrepreneur that markets the city to global investors. The result is an institutionalised form of state–corporate hegemony in which political and economic power converge spatially. Batam’s trajectory also suggests that this ideological configuration can be remarkably durable: the island has functioned on this logic for more than five decades, indicating that “fragmentation” in the Sechi and Golubchikov sense is not necessarily a transitional phenomenon but can constitute a stable regime of urbanisation.

The ideological framing of development also masks the exclusionary and coercive aspects of urban policy. Evictions of informal settlements to clear land for industrial or infrastructural projects are often justified as necessary for modernisation. Meanwhile, the rhetoric of investor confidence and global integration obscures the lived realities of displacement and precarity. Citizens are recast as consumers or economic subjects, their rights subordinated to the imperatives of capital. This depoliticisation of space aligns with what Lefebvre calls the illusion of transparency: the belief that space is neutral, when in fact it embodies and perpetuates social relations of domination.

### Actor interaction and the contradictions of urban governance

The empirical findings reveal that Batam’s urban governance is marked by institutional duality and tension between BP Batam and the Batam City Government. While decentralisation reforms in Indonesia were intended to democratise governance, Batam represents an anomaly in which authority over land and industrial policy remains concentrated in a quasi-centralised agency. The coexistence of parallel bureaucratic structures creates overlapping mandates, inconsistent regulation, and competition for jurisdictional control. The PKB secretary’s joke that the mayor “had only the people” while the authority “had the land” is not merely folk wisdom; it is an accurate functional description of a governance regime in which popular sovereignty and territorial sovereignty have been institutionally separated. That this separation is experienced as legible and even humorous by political insiders tells us how deeply it has been naturalised.

This bureaucratic fragmentation is not merely a technical problem but a manifestation of what Sechi and Golubchikov (2025) identify as the fragmented institutional landscapes of neoliberal urbanism. In such contexts, governance is decentralised only superficially; in practice, decision-making remains centralised within elite networks that align with capital interests. The “contested authority” between BP Batam and the city government thus reflects a deeper contradiction: the simultaneous localisation and centralisation of power under neoliberalism. The state is reconfigured, not weakened, to secure capital’s spatial reproduction while managing the political risks of inequality. BP Batam’s Head of Public Relations was candid that the institutional “clash” between the two bodies was resolved in 2019 not by redistributing authority but by unifying it in a single ex officio official, a solution that accelerated infrastructural investment but further narrowed the channels through which residents could contest spatial decisions.

### Differential space and the everyday politics of survival

Despite the dominance of abstract and fragmented space, Lefebvre insists that resistance persists through differential space, meaning spaces that emerge within and against the contradictions of capitalist spatial order. In Batam, informal settlements (ruli) exemplify this possibility. Although marginalised, precarious, and often criminalised, these settlements represent lived spaces of appropriation and solidarity. Residents construct housing, share resources, and negotiate access to infrastructure, exercising agency in shaping their environment. In doing so, they reclaim use value over exchange value, asserting a right to inhabit the city on their own terms. The Head of BPN’s striking description of ruli as, for some residents, ‘a profession,’ referring to a rotating cycle of eviction, compensation, sale, and re-occupation, refines rather than contradicts the Lefebvrian reading. It demonstrates that ruli are not simply sites outside the commodified city but sites through which its edges are continually renegotiated, as dispossessed inhabitants learn to extract exchange value from their own displacement and then convert it back into the use values of informal dwelling.

These practices constitute a subtle yet potent form of spatial politics. They reveal that even within a highly commodified and regulated urban system, the production of space remains incomplete and contested. Ruli communities, through their improvisational and collective spatial practices, resist total enclosure by the market–state nexus. Their very presence challenges the hegemonic representation of Batam as a sanitised, investor-friendly industrial landscape. In Lefebvrian terms, they inhabit and re-signify the cracks of abstract space, transforming marginality into a site of social production. The Jacobs (1961) insight that planned monofunctional districts often lack the mixed-use vitality that sustains urban social life is visible here in the contrast between the clean but largely empty industrial estates after working hours and the dense, self-organised everyday rhythms of the ruli.

This everyday contestation underscores the dialectical nature of space: it is both a product of domination and a medium for resistance. The production of space in Batam thus cannot be understood solely through the logic of capital or state planning. It must also account for the lived practices that subvert and reconfigure spatial meaning from below. In this sense, Batam illustrates the coexistence of fragmented neoliberal space and emergent differential space, a duality that defines many post-authoritarian, semi-peripheral urban contexts in the Global South.

## Conclusion

This study has shown that the production of space in Batam is not a neutral or technical process but a historically specific and ideologically charged project shaped by the interaction of the state, capital, and planning institutions. Viewed through Lefebvre (1991) theory of the production of space, Batam exemplifies the dominance of abstract space: space conceived and organised for accumulation, efficiency, and control. Following Sechi and Golubchikov (2025), this abstraction manifests today as neoliberal space fragmentation, in which the city is restructured into differentiated, commodified, and privatised enclaves that normalise inequality as a by-product of economic efficiency. The interview material reinforces this reading from within: a senior state official describes the city as founded by business rather than by government, BP Batam’s own communications office recalls institutional “clash” resolved by centralisation rather than by democratisation, and a political insider distils the governance regime into the observation that the mayor has people while the authority has land. These are not external critiques but internal self-descriptions of a fragmented spatial order.

In relation to existing theory, the paper makes three contributions. First, it extends Lefebvrian analysis beyond its conventional European empirical base into a Southeast Asian state-capitalist SEZ, demonstrating that the triadic model remains analytically productive in semi-peripheral contexts provided it is coupled with an account of fragmentation. Second, it shows that Sechi and Golubchikov’s (2025) fragmentation thesis, developed primarily from post-socialist European cases, travels to SEZ-driven urbanisation in the Global South and is strengthened by engagement with long-running state-capitalist cases such as Batam, where fragmentation has proved a stable rather than transitional regime. Third, it refines the concept of differential space by showing that rumah liar are not merely symbolic sites of resistance but are dialectically constitutive of Batam’s abstract space, absorbing labour that the formal SEZ economy requires but refuses to house, and metabolising eviction and compensation into new cycles of informal settlement.

The argument opens several avenues for further research. Comparative studies across Southeast Asian SEZs, for example, Iskandar Malaysia, the Thilawa SEZ in Myanmar, or the Vietnam–Singapore Industrial Park, would test whether the patterns of fragmentation identified here are specific to the Batam–Singapore relationship or generalise across the region. Comparative work with Chinese SEZs such as Shenzhen could illuminate how different state-capitalist configurations produce different spatial orders. Longitudinal research following the trajectories of specific ruli communities over time would extend the analysis beyond the snapshot this study provides, while gendered and generational analyses of informal settlement life could further develop the feminist Lefebvrian agenda initiated by scholars such as Kipfer and colleagues (Goonewardena et al., 2008). Batam’s spatial dynamics ultimately reaffirm Lefebvre’s insight that space is always political, and, as Sechi and Golubchikov (2025) argue, that it remains the key medium through which neoliberal hegemony is constructed, maintained, and potentially undone.

## Data Availability

The original contributions presented in the study are included in the article/supplementary material, further inquiries can be directed to the corresponding author.
